# MAP1S Controls Breast Cancer Cell TLR5 Signaling Pathway and Promotes TLR5 Signaling-based Tumor Suppression

**DOI:** 10.1371/journal.pone.0086839

**Published:** 2014-01-23

**Authors:** Ming Shi, Yuanfei Yao, Fang Han, Yiqun Li, Yu Li

**Affiliations:** School of Life Science and Technology, Harbin Institute of Technology, Harbin, China; The University of Hong Kong, China

## Abstract

Targeting TLR5 signaling in breast cancer represents a novel strategy in cancer immunotherapy. However, the underlying mechanism by which TLR5 signaling inhibits cancer cell proliferation and tumor growth has not been elucidated. In this study, we found TLR5 agonist flagellin inhibited the cell state of activation and induced autophagy, and reported that autophagy protein MAP1S regulated the flagellin/TLR5 signaling pathway in breast cancer cells through enhancement of NF-κB activity and cytokine secretion. Remarkably, MAP1S played a critical role in tumor suppression induced by flagellin, and knockdown of MAP1S almost completely abrogated the suppression of tumor growth and migration by flagellin treatment. In addition, elevated expression of MAP1S in response to flagellin feed-back regulated tumor inflammatory microenvironment in the late stages of TLR5 signaling through degradation of MyD88 in autophagy process. These results indicate a mechanism of antitumor activity that involves MAP1S-controlled TLR5 signaling in breast cancer.

## Introduction

Toll-like receptors (TLRs) play key roles in both the innate and adaptive immune systems through recognition of pathogen associated molecular patterns (PAMPs) and induction of inflammatory responses [1], [2]. These receptors are expressed not only in immune cells but also in epithelial cells, including various cancer cells [3]. Accumulating evidence indicates that TLRs play important roles in cancer progression [4], [5]. Activation of most TLRs promotes inflammation in the tumor microenvironment and mediates tumor cells immune escape [6], [7], [8]. However, recently, a portion of activated TLRs have also been shown to activate the immune system against cancer [9], [10]. Thus, targeting TLRs represents a potential therapeutic strategy in cancer immunotherapy.

TLR5 is expressed highly in some cancer cells, but is not expressed on mouse macrophages and conventional dendritic cells [11], [12]. TLR5 recognizes flagellin and initiates a signaling cascade through recruitment of MyD88 and activation of NF-κB. Recently, we and other groups determined that among TLR ligands, only the TLR5 ligand flagellin can induce TLR signaling in breast cancer cells [12], [13], [14]. Triggering of TLR5 in cancer cells inhibits cancer cell proliferation and elicits strong antitumor activity [12], [15]. TLR5 signaling also exhibits radioprotective activity and improves the radiation efficacy of tumor cells in radiotherapy [16]. However, TLR5 signaling in gastric cancer exhibits the opposite effect [17]. The reason for these different outcomes of TLR5 signaling in different cancers is not clear.

In this study, we focus on the role of MAP1S in TLR5-induced suppression of breast cancer. MAP1S is a recently identified adaptor protein of autophagic processes, which participates in microtubular coordination and regulates autophagy to suppress tumorigenesis [18], [19]. We observed that MAP1S levels were up-regulated in response to flagellin treatment in human breast carcinomas and MAP1S regulated cytokine expression induced by TLR5 signaling. Remarkably, MAP1S was associated with inhibition of cell proliferation and migration of flagellin-treated breast cancer cells. In addition, flagellin-induced elevation of MAP1S expression was involved in inhibitory feedback regulation of TLR5 signaling-induced late stage inflammation through the degradation of MyD88.

## Materials and Methods

### Cell Lines and Reagents

Human breast epithelial cell line MCF-10A, human breast cancer cell lines MCF-7, MDA-MB-435s, MDA-MB-468, T47D, MDA-MB-231 and MDA-MB-431 were originally purchased from the American Type Culture Collection (ATCC, Manassas, VA, USA). Antibodies against MyD88, cyclin D1 and p27Kip1 were purchased from purchased from Abcam. Antibody against MAP1S (4G1) was from Precision Antibody. Antibody against β-actin was purchased from Santa Cruz Biotechnology. Flagellin (FLA-ST) was purchased from InvivoGen. NF-κB and AP-1 luciferase reporter was described previously [20].

### shRNA Transfection and Quantitative RT-PCR

A retroviral-vector based shRNA plasmid was inserted into the retroviral vector pQsupR by combining and ligating the vector and a mixture of oligos encoding shCtrl or shMAP1S digested by Bgl II and Xho I. The sequences of oligos encoding shMAP1S-1, shMAP1S-2 and shCtrl are shown in Table S1. MCF-7 cells were stably transfected with shMAP1S or shCtrl alone using Lipofectamine 2000 and selected with 0.5 µg/ml puromycin (Sigma, St. Louis Mo., USA) for 14 days. Quantitative RT-PCR was conducted as described previously [21]. Primer sequences are shown in Table S1.

### Luciferase Assays and Western Blotting

Cells were seeded in 24-well plates at 50%–60% confluence. The following day, cells were co-transfected with NF-κB or AP-1 luciferase reporters and vector or pcDNA3.1-MyD88, pcDNA3.1-MAP1S, pQsupR-shCtrl or pQsupR-shMAP1S using Lipofectamine 2000 (Invitrogen, Carlsbad, CA, USA). Luciferase activity was measured as described previously [12]. Western blotting was conducted as described previously [21].

### Transwell Co-culture Assay and Transwell Migration Assay

MCF-7/shCtrl and MCF-7/shMAP1S cells were counted and seeded at 40,000 cells per chamber in Transwell inserts with 3.0 µm polycarbonate membranes in 24-well plates. Wild type MCF-7 cells were seeded at 2,000 cells per well into the lower chamber. The cells in upper chamber were pretreated with flagellin for 6 h, rinsed with PBS and supplied with fresh medium. The co-culture Transwell system was incubated at 37°C for 6 days, and the upper chamber with pretreated cells was changed every second day. Cell proliferation was detected every second day by the MTT assay. A Transwell migration assay was conducted as described previously [22].

### Immunofluorescence Staining

Immunofluorescence analysis was performed on MCF-7 cells as described previously [22]. Briefly, MCF-7 cells were grown on coverslips in Dulbecco’s modified Eagle medium sup-plemented with 10% fetal bovine serum for 24 h (approximately 60% confluence). Cells were fixed with 4% paraformaldehyde in PBS buffer (pH 7.4) for 20 min at RT and rinsed with PBS. The coverslips were then mounted and visualized using a Zeiss LSM 510 META con-focal microscope. Images were taken employing Zeiss LSM software.

### MTT Proliferation Assay and Cell Cycle Analysis

Cells were seeded into 96-well plates at 2.0×10^3^ cells per well and exposed to 0.5 µg/ml flagellin for the indicated number of days. The cells were then incubated in 0.5 mg/ml MTT solution at 37°C for 4 hours, and 100 µl of detergent reagent was added to the wells. After overnight incubation at 37°C, absorbance at 490 nm was measured with a microplate spectrophotometer (Infinite M200; TECAN, Salzburg, Austria). Cell cycle analysis was conducted as described previously [12].

### Whole-cell MALDI-TOF MS

Whole-cell MALDI-TOF MS was conducted as reported previously [23]. Briefly, cell samples were dissolved in a α-cyano-4-hydroxy-cynnamique acid (CHCA) or 2, 5-dihydroxybenzoic acid (DHB) matrix and then dried on a MALDI target. The fingerprint was detected and analyzed using an Autoflex III mass spectrometer and FlexAnalysis software (Bruker Daltonics, Bremen, Germany).

### Cell Scratch Healing Assay and Soft Agar Colony Assay

Stable MCF-7/shCtrl or MCF-7/shMAP1S cells were seeded in duplicate in a 12-well culture plate and grown to confluence. A scratch wound was applied in the confluent cell-layer with a sterile 20 µl pipette tip. Wounds were observed by phase-contrast microscopy. The wound width was compared with the initial width in different fields from three independent experiments. The soft agar colony assay was conducted as described previously [12].

## Results

### Autophagic Factor MAP1S Links with TLR5 Pathway in Breast Cancer Cells

**Figure 1:**
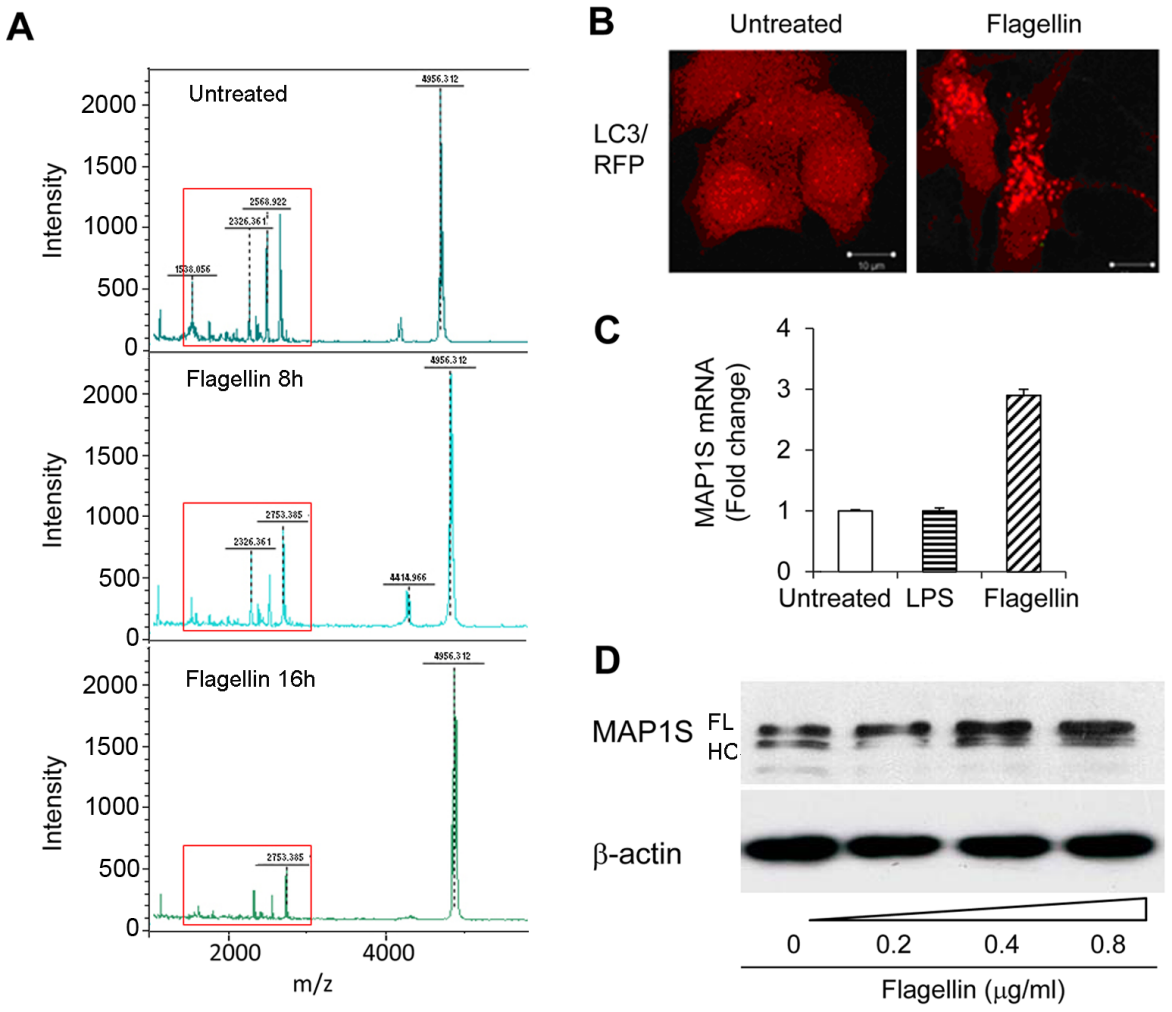
MAP1S links with cancer cell TLR5 signaling pathway. (A) Whole-cell MALDI-TOF MS spectrum of MCF-7. MCF-7 cells were stimulated with 0.5 µg/ml flagellin for 16 h. Then cells were collected in 10 µl of PBS, and 1 µl suspension was mixed with CHCA matrix and loaded onto MALDI target. (B) MAP1S promotes TLR signaling-induced autophagy. LC3 puncta induced by flagellin in MCF-7 cells were detected by immunofluorescence. Magnification, 63×. (C) MAP1S mRNA expression in untreated, LPS (0.1 µg/ml) or flagellin (0.1 µg/ml) treated MCF-7 cells was detected by quantitative RT-PCR. (D) MCF-7 cells were stimulated by the indicated doses of flagellin for 16 h. MAP1S protein levels were analyzed by western blotting. All data are representative results of 3 independent experiments.

TLR5 signaling can be induced by flagellin in MCF-7 cells [12]. To globally analyze the cell state of activation in flagellin-treated MCF-7 cells, we performed the whole-cell MALDI-TOF MS [23], [24]. The spectra of untreated MCF-7 and flagellin-treated MCF-7 cells were clearly different. The fingerprint of MCF-7 was specific, and peaks ranging from 1000 to 3000 m/z were significantly lowered under flagellin treatment (Figure 1A), while LPS only induced little response (Figure S1A). 3 specific peaks (m/z = 1538, 2568, and 2753) were identified to characterize the response of MCF-7 to flagellin, and the intensity of the specific peaks was significantly decreased to 23.9%, 35.5%, and 50.6% respectively after 16 h of flagellin treatment (Figure S1B). Since different matrix would produce different MALDI mass spectra, 2 kinds of matrix (CHCA and DHB) were used in the whole-cell MALDI-TOF MS. There existed different peaks from the range of 1000 to 3000 m/z in CHCA and DHB matrix-applied MALDI MS, but both spectra showed similar phenomena when MCF-7 cells treated with flagellin (Figure S1C). It indicated that TLR5 signaling induced by flagellin inhibited the cell state of activation. Because TLR activation can induce autophagy [25], which is a homeostatic, catabolic degradation process and inducer of state of dormancy in cancer cells [26], we wonder whether flagellin can induce autophagy in breast cancer cells. Indeed, flagellin can induce LC3 foci, a marker of autophagy, in breast cancer MCF-7 cells (Figure 1B). We then wanted to determine whether autophagy can regulate the TLR5 signaling pathway in breast cancer cells. MAP1S is a recently identified autophagy protein [19], [27]. We found that MAP1S is ubiquitously expressed in the breast epithelial cell line MCF-10a and breast cancer cell lines including MCF-7, MDA-MB-435s, MDA-MB-468, T47D, MDA-MB-231 and MDA-MB-431 (Figure S2).

Next, MAP1S expression was detected by real-time PCR. Its expression was up-regulated in response to flagellin treatment, but did not respond to LPS (Figure 1C). We also confirmed MAP1S expression was elevated under flagellin treatment in dose dependent way by western blotting. MAP1S full length (FL) and heavy chain (HC) were the main products in MCF-7 cells, and MAP1S FL showed significant increase in response to flagellin treatment (Figure 1D). Collectively, these results indicated that MAP1S is associated with breast cancer TLR5 pathway.

### MAP1S is Required for Flagellin to Inhibit Breast Cancer Cell Proliferation

**Figure 2:**
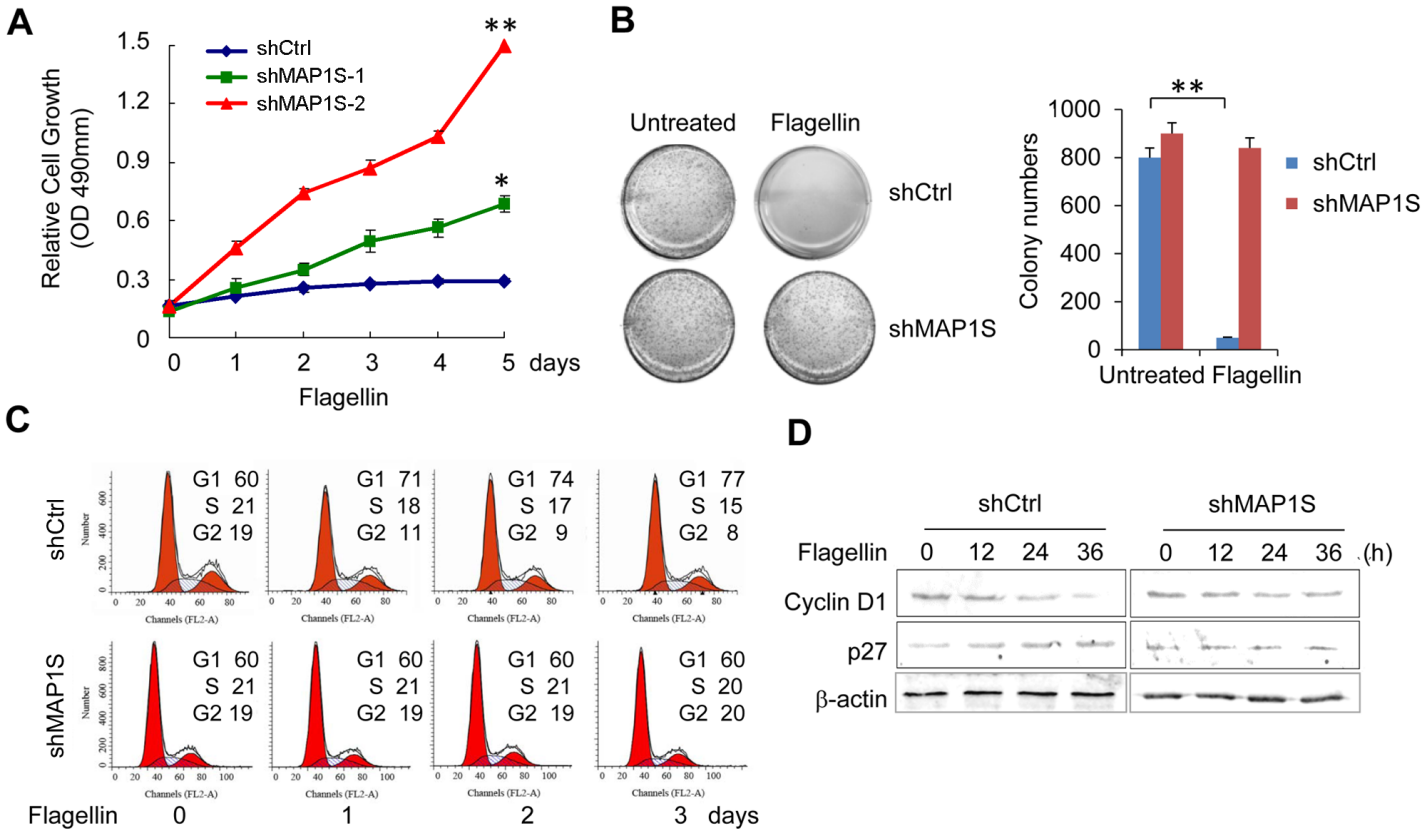
MAP1S is required for flagellin to inhibit breast cancer cell proliferation. (A) The cell proliferation of MCF-7/shCtrl and MCF-7/shMAP1S cells was determined using the MTT assay after treatment with 0.5 µg/ml flagellin for the indicated number of days. (B) Four representative 35-mm plates of MCF-7/shCtrl and MCF-7/shMAP1S-2 cells were cultured for 8 days with or without flagellin treatment. Cells were then stained with 1 mg/ml MTT solution for 30 min and images of the colonies that formed were captured with a microscope. The number of colonies (≥100 µm in diameter) was counted using Image-Pro Plus software. **indicates *p*<0.001, relative to untreated cells. (C) MCF-7/shCtrl and MCF-7/shMAP1S-2 cells were stimulated with 0.5 µg/ml flagellin for the indicated number of days. DNA content was assessed by PI staining and flow cytometry. Quantitative data showing different phases of the cell cycle is displayed. (D) MCF-7/shCtrl and MCF-7/shMAP1S-2 cells were treated with 0.5 µg/ml flagellin for the indicated time periods, and Cyclin D1 and p27 level were analyzed by western blotting with specific antibodies. All data are representative results of 3 independent experiments.

Our previous report showed that the TLR5 agonist flagellin has a strong capacity to inhibit tumor growth [12]–[14]. We therefore analyzed the role of MAP1S in cell proliferation after flagellin treatment using the MTT assay. MAP1S was knocked down in MCF-7 by transfection with retroviral vector/shMAP1S, and mRNA and protein level of MAP1S in MCF-7 knocked down cells were tested by real time PCR and western blot (Figure S3). The MTT assay showed that deletion of MAP1S abrogated the growth inhibition of MCF-7 cells following flagellin treatment. Lower expression of MAP1S made cells more resistant to the antitumor growth effects of flagellin (Figure 2A). A single-cell clone formation assay in soft agar was also used to evaluate the role of MAP1S in the anchorage-independent growth of MCF-7 cells and the cells’ response to flagellin. The antitumor effect of flagellin on MCF-7 cells was reduced by knockdown of MAP1S. As shown in Figure 2B, there were many cell clones formed in untreated groups, while not a single clone formed in flagellin treated MCF-7/shCtrl cells. Cells transfected with ShMAP1S showed very little response to flagellin treatment. Furthermore, the cell cycle of MCF-7/shCtrl cells and MCF-7/ShMAP1S was measured. G1 arrest was observed in flagellin treated MCF-7/shCtrl cells, but not in MAP1S knockdown cells (Figure 2C). To investigate the molecular mechanism of the observed effects of MAP1S on MCF-7 cells response to flagellin, we analyzed the expression level of cell cycle proteins cyclin D1 and CDK inhibitor p27. We found that cyclin D1 levels were significantly decreased and p27 expression was increased in flagellin-stimulated MCF-7/shCtrl cells, while no obvious changes in cyclin D1 and p27 levels were observed in flagellin treated MCF-7/ShMAP1S cells (Figure 2D). Therefore, in addition to autophagy, G1 arrest induced by flagellin treatment is another reason for MAP1S-involved inhibition of breast cancer cell proliferation.

### MAP1S Regulates Expression of Flagellin-induced Cytokines and Soluble Factors in Breast Cancer Cells

**Figure 3:**
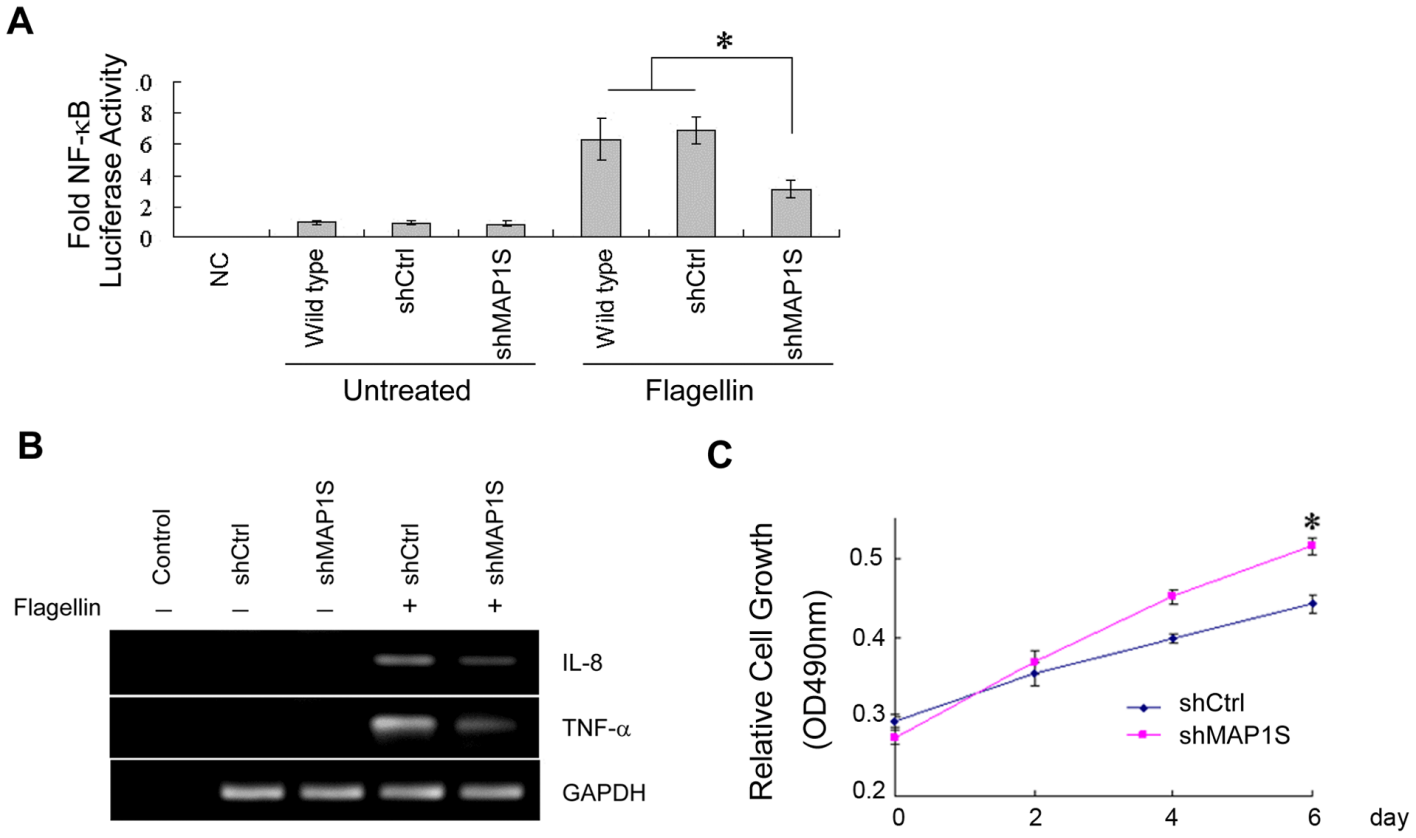
MAP1S regulates flagellin-induced cytokines and soluble factors in breast cancer cells. (A) Wild type MCF-7, stable MCF-7/shCtrl and MCF-7/shMAP1S cells were transfected with NF-κB reporter plasmids and analyzed for luciferase activity induced by flagellin. *indicates *p*<0.05, relative to flagellin-treated wild type and shCtrl cells. NC, negative control. (B) MCF-7 cells were stimulated with 0.1 µg/ml flagellin for 6 h for cytokine expression. The mRNA levels were analyzed by quantitative RT-PCR. (C) Wild type MCF-7 cells co-cultured with flagellin-pretreated MCF-7/shCtrl or MCF-7/shMAP1S cells in a co-culture Transwell system. Cell proliferation on the indicated day was detected by the MTT assay. *indicates *p*<0.05, relative to shCtrl cells.

Although TLR5 has been shown to activate the NF-κB signaling pathway and induce cytokine expression, the regulatory mechanisms involved in TLR5 signaling have not been fully elucidated. To this end, we tested the effect of MAP1S on TLR5 signaling-induced NF-κB activity in flagellin-stimulated MCF-7 cells using an NF-κB luciferase assay. Flagellin-induced NF-κB activity was significantly decreased in MCF-7/ShMAP1S cells compared with wild type and shCtrl controls (Figure 3A). To further explore the role of MAP1S in TLR5-induced cytokine expression, we measured the relative mRNA levels of IL-8 and TNF-α in untreated or flagellin treated MCF-7/shCtrl or MCF-7/ShMAP1S cells. As shown in Figure 3B, flagellin induced high expression of IL-8 and TNF-α in MCF-7/shCtrl cells, while there was no detectable cytokine expression in the untreated control. Importantly, MAP1S deletion led to decreased cytokine expression in flagellin treated MCF-7/ShMAP1S cells compared to flagellin treated MCF-7/shCtrl cells.

Our previous results showed that the soluble factors induced by flagellin in MCF-7 cells inhibited the proliferation of cancer cells [12]. Here, we tested the role of MAP1S in tumor suppression induced by these soluble factors in a co-culture Transwell system. The data demonstrated that knockdown of MAP1S blocked the flagellin-induced growth inhibition by soluble factors (Figure 3C), which suggested that MAP1S also facilitates the expression of soluble factors induced by flagellin.

### Overexpressed MAP1S Inhibits Tumor Cell TLR5 Signaling

**Figure 4:**
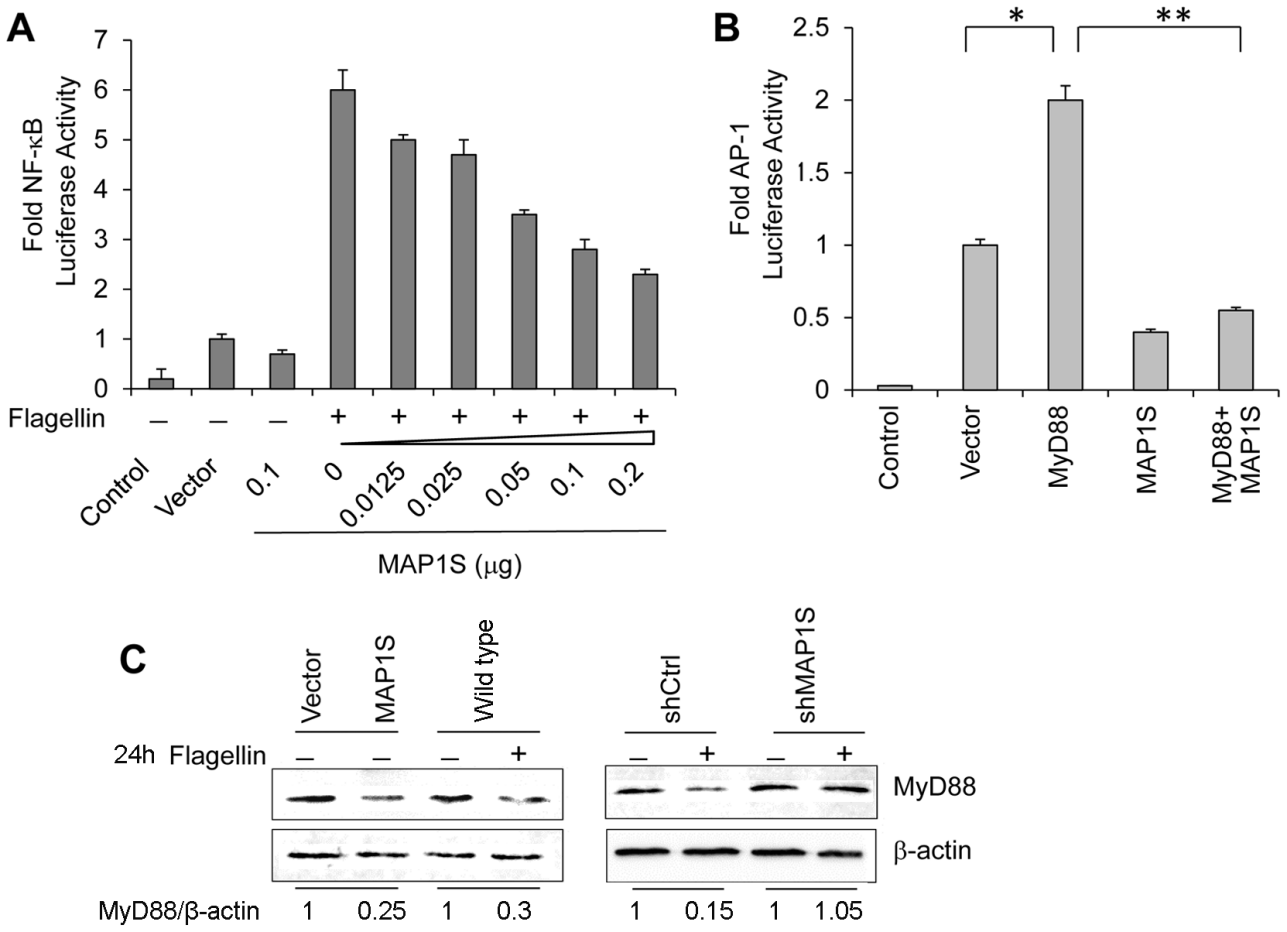
Overexpressed MAP1S inhibits tumor cell TLR5 signaling. (A) MCF-7 cells were transfected with different doses of MAP1S plasmid and NF-κB reporter plasmid and analyzed for luciferase activity induced by flagellin. (B) MCF-7 cells were transfected with AP-1 reporter plasmid, vector control, MyD88 and/or MAP1S and analyzed for luciferase activity. *indicates *p*<0.05, relative to vector control. **indicates *p*<0.001, relative to co-transfection of MyD88 and MAP1S. (C) Left, MCF-7 cells transfected with empty vector or MAP1S expressing plasmid, or wild type MCF-7 cells were treated with 0.5 µg/ml flagellin or vehicle for 24 h. Right, MCF-7/shCtrl and MCF-7/shMAP1S cells were treated with 0.5 µg/ml flagellin or vehicle for 24 h. MyD88 levels were analyzed by western blotting.

To assess the biological function of up-regulated MAP1S expression following flagellin stimulation of TLR5, we transfected MCF-7 cells with different doses of a MAP1S expressing plasmid or a control vector and measured NF-κB activity after flagellin treatment using the luciferase assay. Intriguingly, higher doses of MAP1S plasmids induced lower NF-κB activity in a dose-dependent manner (Figure 4A). Another biological signal that is similar to flagellin stimulation is the well-known activated TLR4, CD4-TLR4, which stimulates NF-κB activity in HEK293 cells. Overexpression of MAP1S also suppressed NF-κB activity in HEK293T/CD4-TLR4 cells (Figure S4). Considering that the key adaptor protein MyD88 played central roles in different TLRs signaling, we transfected MCF-7 cells with MyD88 and/or MAP1S expressing plasmids and tested AP-1 activity using the AP-1 luciferase assay. Transfection of MyD88 expressing plasmids alone enhanced AP-1 activity, while transfection of MAP1S plasmids alone decreased AP-1 activity. Co-transfection of MyD88 and MAP1S plasmids led to no up-regulation of AP-1 activity (Figure 4B). Western blot analysis further indicated that MyD88 was down-regulated following flagellin-stimulated TLR5 activation or overexpression of MAP1S, while no significant change in MyD88 levels was observed in flagellin treated MCF-7/ShMAP1S cells compared with untreated cells (Figure 4C). Taken together, these results indicated that MyD88-mediated signaling was inhibited by elevated MAP1S; therefore, we reasoned that MAP1S might mediate MyD88 degradation in late stages of the TLR5 signaling-induced inflammatory response.

### MAP1S is Essential for Flagellin to Inhibit Tumor Cell Migration

**Figure 5:**
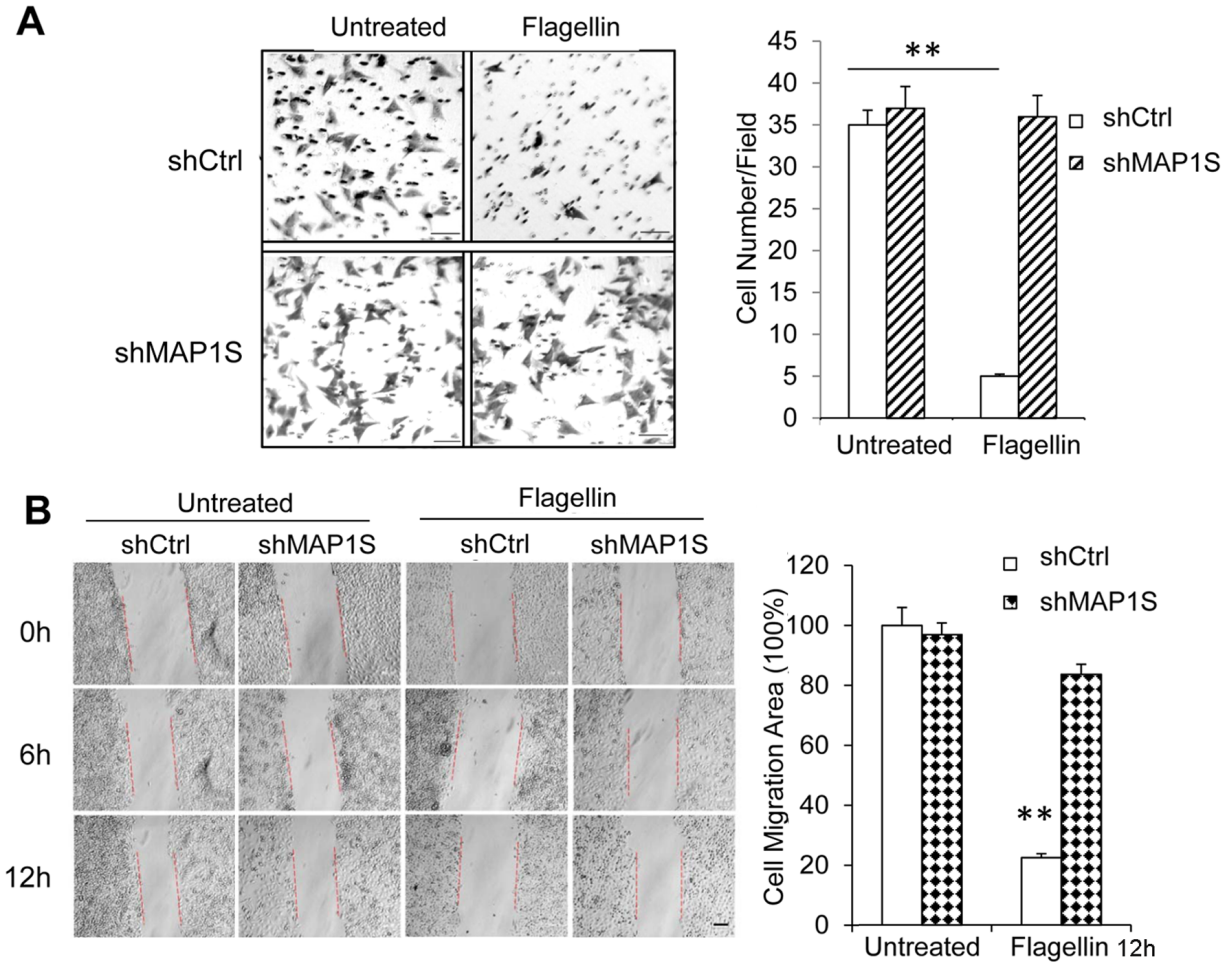
MAP1S is essential for flagellin to inhibit tumor cell migration. (A) Left, MCF-7/shCtrl and MCF-7/shMAP1S cells were seeded into the upper chamber of a Transwell insert and treated with 0.5 µg/ml flagellin for 20 h. Cell migration was measured using a Transwell migration assay. Cells were stained with 0.1% crystal violet after nonmigrated cells were scraped on the upper surface of the polycarbonate membrane. Magnification, 200×. Right, quantitative data on cell migration is shown. (B) Left, MAP1S promoted the inhibition of MCF-7 cell migration by flagellin treatment as measured by a scratch wound migration assay. Healing due to cell migration was observed over a period of 6 h and 12 h following scratch wounding. Magnification, 10×. Right, quantitative data from the cell scratch healing assay is shown. The difference in migratory capacity was significant in flagellin-treated MCF-7/shCtrl cells compared with untreated cells and flagellin-treated MCF-7/shMAP1S cells. **indicates *p*<0.001, relative to untreated cells.

Considering the function of MAP1S in microtubule dynamics [28], we further analyzed the role of MAP1S in the migration capacity of flagellin treated MCF-7 cells using the Transwell migration assay. We found that flagellin almost completely blocked the migration ability of MCF-7 cells with shCtrl. Interestingly, MAP1S deletion rescued the migration capacity of flagellin-treated MCF-7 cells (Figure 5A). To confirm the effect of MAP1S on the migration potential of flagellin-treated MCF-7 cells, we did cell migration-scratch healing assay. As shown in Figure 5B, flagellin inhibited cell migration by up to ∼80% at 12 h compared with the untreated shCtrl control. MAP1S knockdown did not alter the ability of cells to migrate, but altered the response to flagellin treatment. Flagellin only slightly decreased cell migration in MCF-7/ShMAP1S cells. Thus, MAP1S controls the TLR5 signaling pathway in cancer cells.

## Discussion

Increasing evidence suggests that TLR5 signaling may play a role in tumorigenesis [12], [15]. Our previous results also showed that activation of TLR5 by flagellin elicited strong antitumor activity in breast cancer cells [12]. In this study, we further investigated the underlying antitumor mechanisms of TLR5 signaling in breast cancer cells by examining the function of MAP1S. MAP1S is an important autophagic adaptor and is linked with suppression of tumorigenesis through the regulation of autophagy [27]. We found that MAP1S levels are elevated in response to flagellin stimulation in MCF-7 cells, but it does not responded to LPS treatment. Flagellin-treated MCF-7 cells exhibited increases in the number of LC3 punctate foci, an autophagy marker (Figure 1A). MCF-7 cells transfected with CD4/TLR4 or MAP1S alone showed few LC3 foci, while transfection of both plasmids induced larger numbers of foci (Figure S5), suggesting that MAP1S enhances TLR signaling-induced autophagy. Furthermore, knockdown of MAP1S dramatically decreased expression of the cytokines IL-8 and TNF-α and decreased NF-κB activity induced by TLR5 signaling. Taken together, these observations indicate that MAP1S is an autophagic regulator involved in TLR5 signaling in breast cancer cells.

Consistent with our previous reported results [12], we demonstrated that flagellin suppressed proliferation of breast cancer cells. Furthermore, the whole-cell MALDI-TOF MS assay showed that flagellin inhibited MCF-7 cell state of activation globally (Figure 1A). We also found MAP1S played a critical role in tumor suppression induced by flagellin treatment. Knockdown of MAP1S almost completely abrogated the inhibition of tumor growth and migration by flagellin treatment, which is consistent with a previous report showing that MAP1S deficient mice frequently develop tumors [27]. We observed G1/S arrest, a significant decrease of cell cyclin protein CyclinD1 and increased p27 levels in MCF-7/shCtrl cells upon flagellin treatment, while there were no obvious changes in CyclinD1 and p27 levels in flagellin-treated MCF-7/shMAP1S cells. These results indicated MAP1S played an important role in antitumor activity of flagellin/TLR5 signaling in MCF-7 cells.

MAP1S enhances autophagic activity and is induced by stress exposure, indicating autophagic activation [19]. In agreement with this study, we observed that elevated MAP1S levels were accompanied by degradation of MyD88 and attenuation of MyD88-dependent transcription factor activation, suggesting that MAP1S provides negative feedback regulation in the TLR5 signaling pathway of breast cancer cells. Unlike starvation, TLR signaling induction of autophagy was a delayed response, which required approximately 16 hours [29], [30]. We also found that MAP1S promoted TLR5 signaling in the early stages of flagellin stimulation through enhancement of NF-κB activity and cytokine expression. In the late stage of inflammation (12–24 hours after flagellin treatment), elevated MAP1S enhanced autophagy and negatively regulated TLR5 signaling-induced transcription factor activation through degradation of MyD88. This enhanced autophagy might further suppress tumorigenesis [31].

In this study, we showed that MAP1S acted as a critical regulator in the antitumor activity of TLR5 signaling in breast cancer cells. In the early stage of TLR5 activation, MAP1S modulated the production of proinflammatory cytokines and unknown soluble factors to elicit potent antitumor activity in breast carcinomas. In the late stage of TLR5-induced inflammation, elevated MAP1S negatively regulated the tumor microenvironment to inhibit inflammation and induced autophagy to suppress tumorigenesis.

## Supporting Information

Figure S1 **Whole-cell MALDI-TOF MS spectrum of MCF-7 treated with LPS or flagellin.** MCF-7 cells were treated with 0.1 µg/ml LPS or flagellin for 0 or 16 h. Then cells were collected in 10 µl of PBS, and 1 µl suspension was mixed with CHCA (A) or DHB (C) matrix to load onto MALDI target. (B) Specific peaks in whole-cell MALDI-TOF MS were identified to characterize the response of MCF-7 to flagellin. Statistical analysis was performed using the student t test. *, indicated *p*<0.05.
(TIF)

Figure S2 **MAP1S is ubiquitously expressed in breast epithelial cell line and breast cancer cell lines.** MAP1S mRNA of 7 breast cell lines was analyzed by semi-quantitative RT-PCR. 1, MCF-10a; 2, MCF-7; 3, MDA-MB-435S; 4, MDA-MB-468; 5, T47D; 6, MDA-MB-231; 7, MDA-MB-431.
(TIF)

Figure S3 **Generation of MAP1S knockdown cells.** MAP1S knockdown effect in MCF-7/shMAP1S was detected by semi-quantitative RT-PCR and western blotting.
(TIF)

Figure S4 **Overexpression of MAP1S suppressed NF-κB activity in HEK293T/CD4-TLR4 cells.** HEK293T or stable HEK293T/CD4-TLR4 cells were co-transfected with NF-κB reporter plasmids and MAP1S expressing plasmid, and then analyzed for luciferase activity.
(TIF)

Figure S5 **Co-transfection of MAP1S and CD4-TLR4 induced LC3 foci in MCF-7 cells.** Stable MCF-7/LC3 cells were co-transfected with MAP1S and CD4-TLR4 expressing plamids. Induction of LC3 foci in MCF-7 cells were detected by immunofluorescence.
(TIF)

Table S1 **oligo sequences.**
(TIF)
