# Supplementary figures and images for: MAP1S Controls Breast Cancer Cell TLR5 Signaling Pathway and Promotes TLR5 Signaling-based Tumor Suppression
Source: PLoS One. 2014 Jan 23;9(1):e86839. doi: 10.1371/journal.pone.0086839 (PMC3900661; doi:10.1371/journal.pone.0086839)

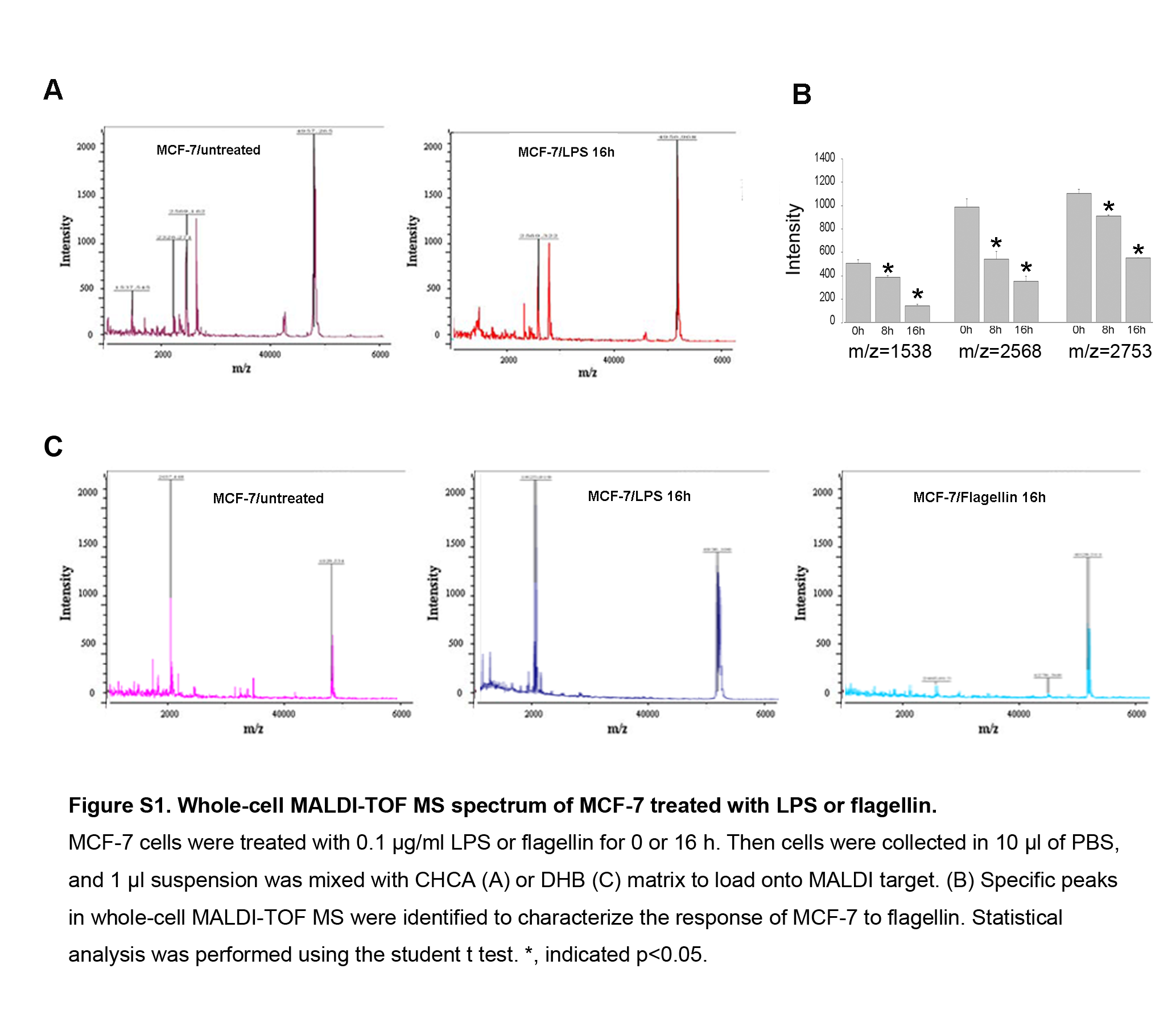

Supplement: Figure S1 — Whole-cell MALDI-TOF MS spectrum of MCF-7 treated with LPS or flagellin. MCF-7 cells were treated with 0.1 µg/ml LPS or flagellin for 0 or 16 h. Then cells were collected in 10 µl of PBS, and 1 µl suspension was mixed with CHCA (A) or DHB (C) matrix to load onto MALDI target. (B) Specific peaks in whole-cell MALDI-TOF MS were identified to characterize the response of MCF-7 to flagellin. Statistical analysis was performed using the student t test. *, indicated p<0.05. (TIF) [file pone.0086839.s001.tif]

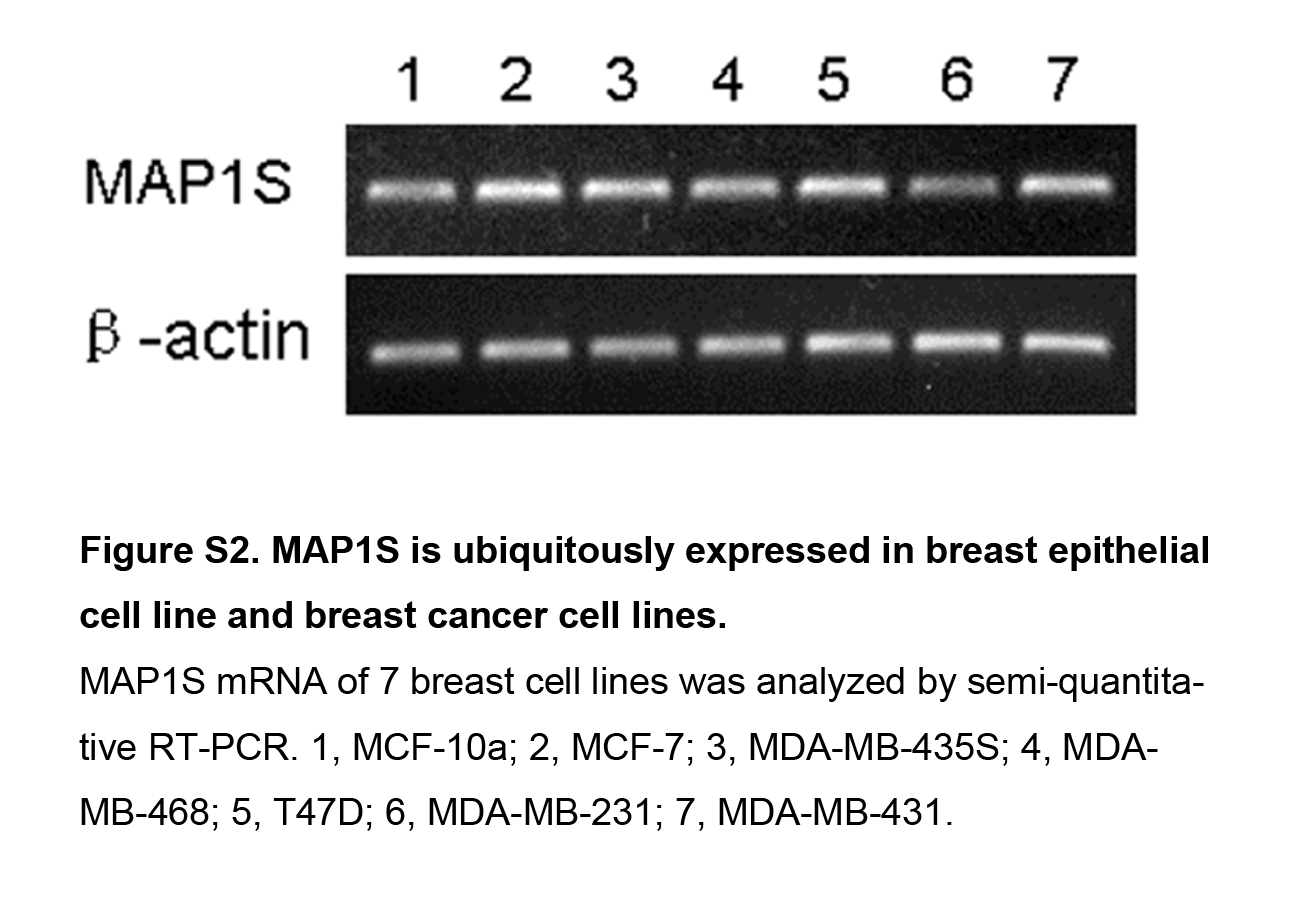

Supplement: Figure S2 — MAP1S is ubiquitously expressed in breast epithelial cell line and breast cancer cell lines. MAP1S mRNA of 7 breast cell lines was analyzed by semi-quantitative RT-PCR. 1, MCF-10a; 2, MCF-7; 3, MDA-MB-435S; 4, MDA-MB-468; 5, T47D; 6, MDA-MB-231; 7, MDA-MB-431. (TIF) [file pone.0086839.s002.tif]

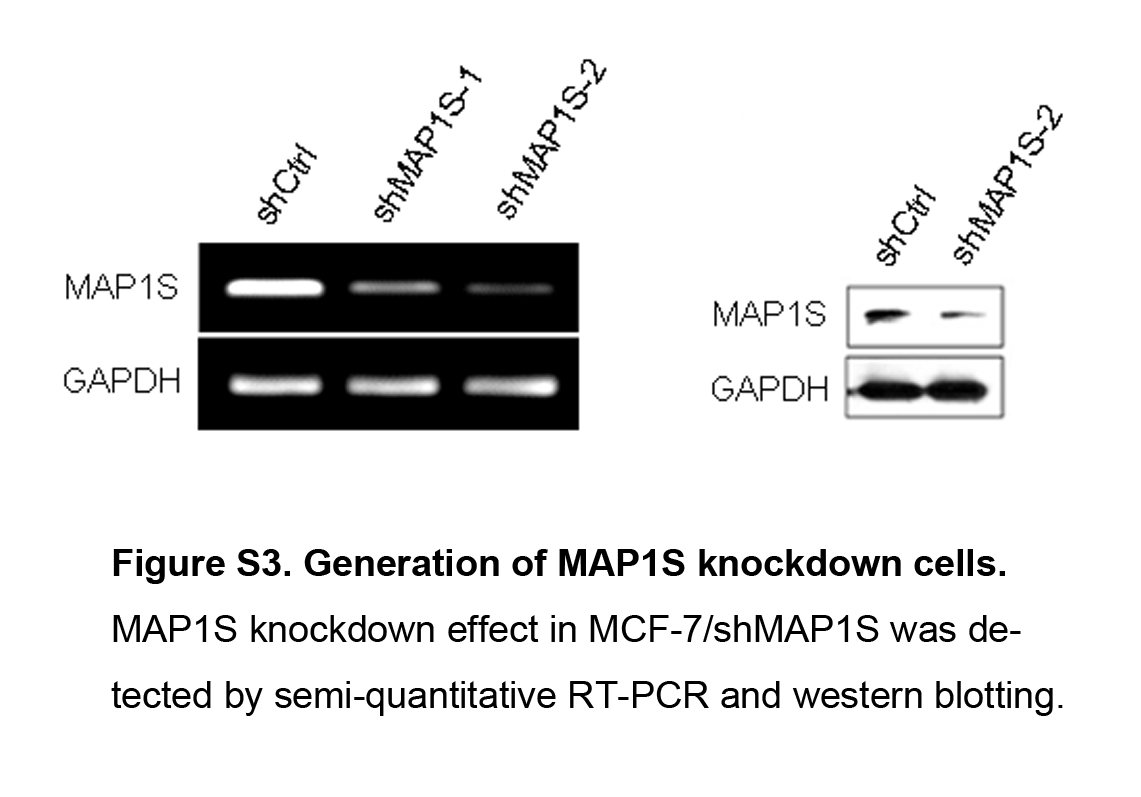

Supplement: Figure S3 — Generation of MAP1S knockdown cells. MAP1S knockdown effect in MCF-7/shMAP1S was detected by semi-quantitative RT-PCR and western blotting. (TIF) [file pone.0086839.s003.tif]

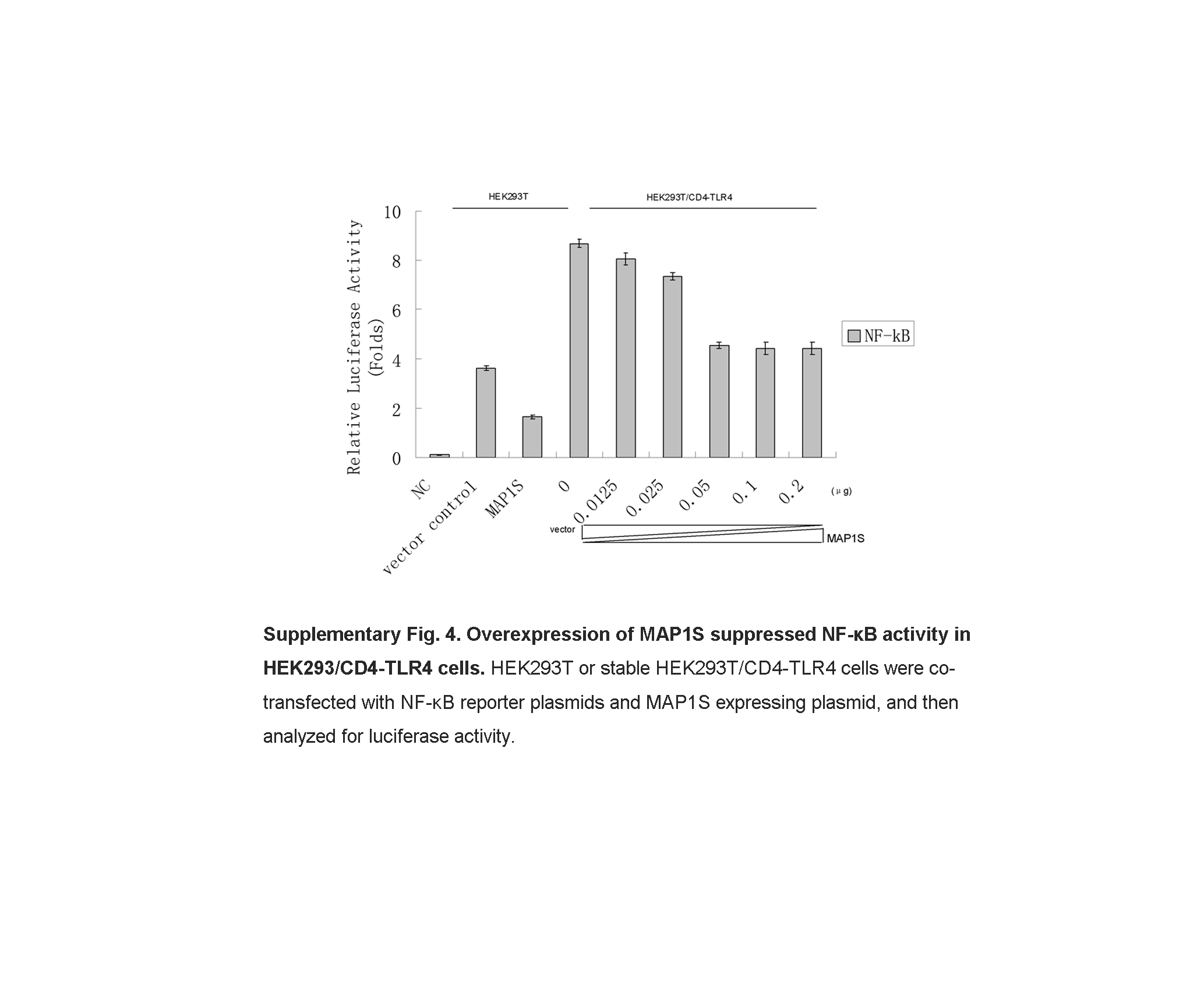

Supplement: Figure S4 — Overexpression of MAP1S suppressed NF-κB activity in HEK293T/CD4-TLR4 cells. HEK293T or stable HEK293T/CD4-TLR4 cells were co-transfected with NF-κB reporter plasmids and MAP1S expressing plasmid, and then analyzed for luciferase activity. (TIF) [file pone.0086839.s004.tif]

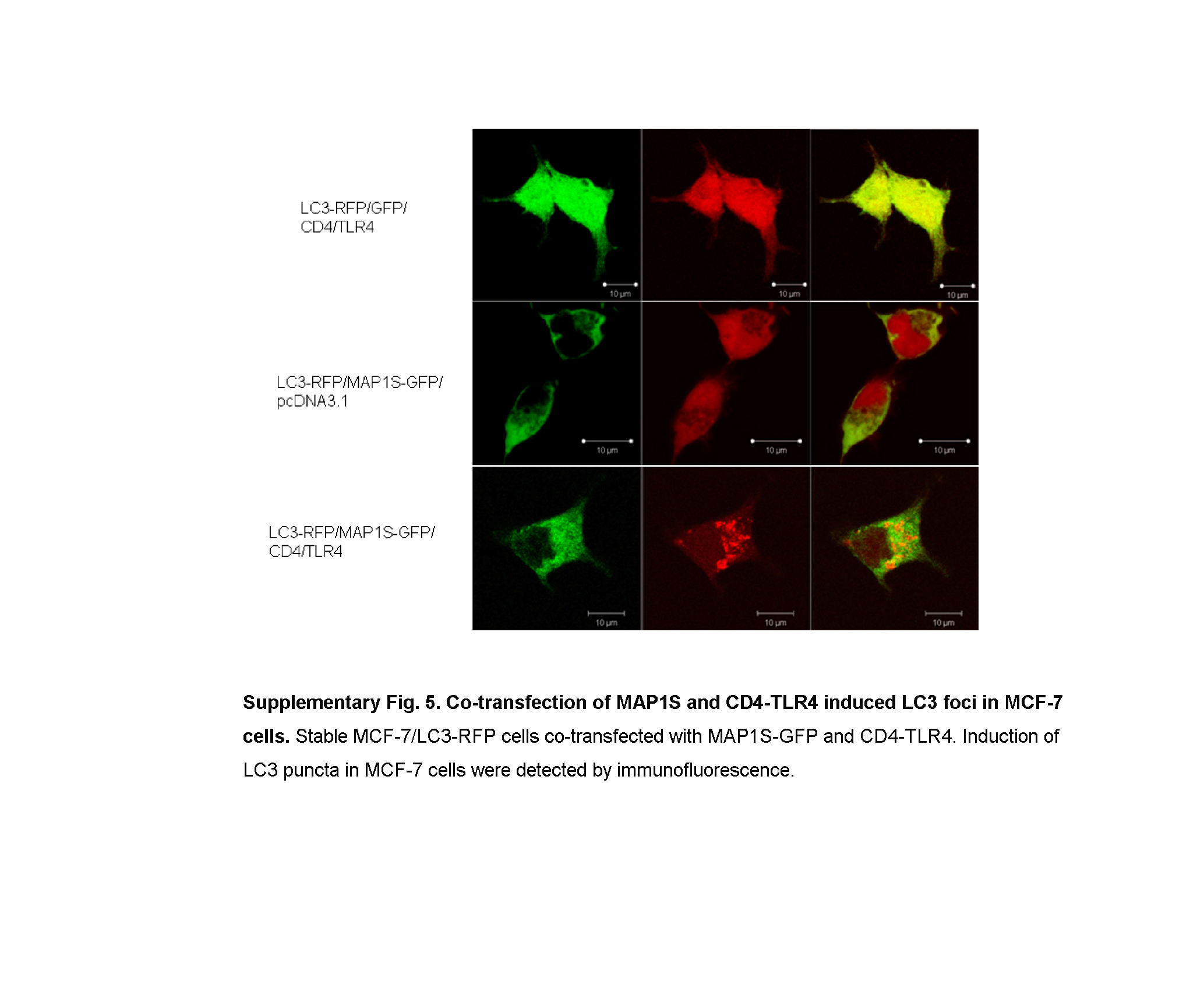

Supplement: Figure S5 — Co-transfection of MAP1S and CD4-TLR4 induced LC3 foci in MCF-7 cells. Stable MCF-7/LC3 cells were co-transfected with MAP1S and CD4-TLR4 expressing plamids. Induction of LC3 foci in MCF-7 cells were detected by immunofluorescence. (TIF) [file pone.0086839.s005.tif]

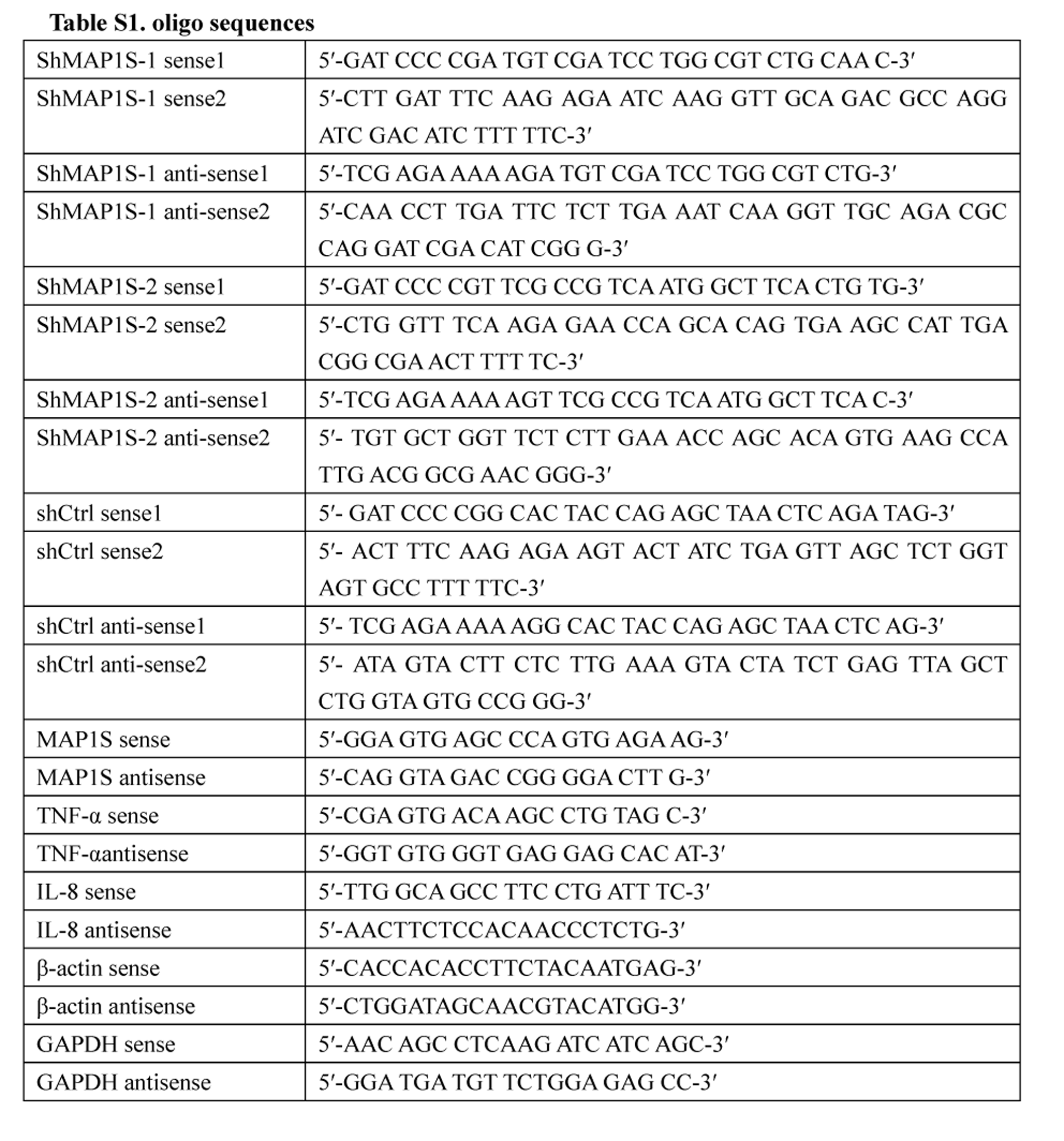

Supplement: Table S1 — oligo sequences. (TIF) [file pone.0086839.s006.tif]
